# Using Social Network Analysis to Inform Implementation Science Infrastructure Development

**DOI:** 10.1007/s43477-025-00180-8

**Published:** 2025-07-31

**Authors:** Stephanie P. Brooks, Reza Yousefi Nooraie, Sara Mortaz Hejri, Denise Thomson, Sara N. Davison, Kate Storey

**Affiliations:** 1 University of Alberta School of Public Health, Edmonton, AB Canada; 2 University of Rochester, Rochester, NY USA; 3 McGill University, Montreal, QC Canada; 4 Alberta SPOR SUPPORT Unit, Edmonton, AB Canada; 5 Department of Medicine, University of Alberta, Edmonton, AB Canada

**Keywords:** Social network analysis, Implementation science, Implementation infrastructure, Capacity building, Collaboration

## Abstract

Implementation is an inherently collaborative and transdisciplinary activity; however, engaging key partners across research, practice, and policy sectors is challenging. Successful implementation requires supportive infrastructure for both research and practice. This paper presents practice-based reflections on the value of exploratory social network analysis during the early phases of developing implementation infrastructure in Alberta, Canada. Specifically, we argue that exploratory social network analysis, when paired with follow-up qualitative interviews, can help identify local implementation science assets, inform network-building, and promote implementation support services to target users. Exploratory social network analysis helped our team identify key implementation researchers and implementation support practitioners in Alberta’s health-research ecosystem. The analysis also showed that implementation research in the province of Alberta follows a consultation model, with one-way assistance requests, while implementation practice is more collaborative in nature. The follow-up interviews provided an opportunity to engage with teams across the networks and allowed participants to contextualize the social network analysis findings. This uncovered: (1) widespread need for implementation science capacity-building, and (2) key implementation partnership considerations. These results illustrate how organizations can employ social network analysis in practical ways to inform implementation infrastructure development.

**Supplementary Information:**

The online version contains supplementary material available at 10.1007/s43477-025-00180-8.

Implementation science is increasingly valued as a means to strengthen innovation uptake in healthcare settings. Specifically, implementation science improves our understanding of change methods, i.e. learning how to put new interventions, policies, or processes into real-world practice (Curran, 2020). Implementation infrastructure is required to implement and sustain evidence-based interventions. Such infrastructure includes animate and inanimate structures and resources (e.g., people power, data monitoring systems, equipment, funding, accountability frameworks) that enable implementation, scale, spread, and sustainment of change. Importantly, implementation infrastructure spans research-to-practice phases, from evidence-based intervention development to scale. Early phase infrastructure supports implementation research to create knowledge around uptake of evidence and change. This knowledge informs and strengthens implementation practice; however, the uptake and sustainment of change requires additional infrastructure to monitor and adapt implementation as needed.

Reporting is limited, but initial studies have explored three ways of framing implementation infrastructure: implementation practice infrastructure, implementation research infrastructure, and integrated infrastructure that facilitates dialogue between research and practice. Evidence shows that implementation practice infrastructure improves the uptake of evidence-based practices and increases how comprehensively new practices are implemented (Brown et al., 2014; Guise et al., 2018; Chambers et al., 2016, Oh 2023). Similarly, implementation research infrastructure enables research to be embedded into rigorous evaluation of real-world implementation (Zullig et al., 2023). Others argue that we should treat practical and research infrastructure as one integrated infrastructure, rooted in research-practice partnerships, that facilitates conducting implementation research that is directly relevant to the host organization’s implementation needs (Brooks et al., 2024a; Oh et al., 2023). This integrated infrastructure is an important component for sustaining change (Oh et al., 2023).

For all three ways implementation infrastructure has been framed, existing studies highlight important infrastructure components required to conduct implementation research and use IS-informed practices. For example, authors agree that a key integrated infrastructure feature is creating embedded implementation research opportunities that achieve shared goals of creating implementation knowledge (Zullig et al., 2023; Oh et al., 2023). Some authors promote cross-organizational implementation research-practice collaborations as an effective integrated infrastructure model (Flynn et al., 2021; Brooks et al., 2024a; Ezeanoule et al., 2018; Sturke et al., 2014). These collaborations assist in contexts where program delivery is not possible through a single agency, necessitating cross-organization partnership (Bryson et al., 2015; Anderson et al., 2023). Data and funding infrastructure supports implementation, scale, and spread (Kilbourne et al., 2020; National Heart Lung & Blood Institute, n.d.; National Institutes of Health n.d., Canadian Institutes for Health Research, 2023; Alberta Health Services, 2023; Oh et al., 2023). Some funding also includes implementation research training components (e.g., National Institutes of Health National Center for Advancing Translational Sciences Clinical and Translational Science Award Programs). All these implementation-strengthening mechanisms are characterized by collaboration. Specifically, these different infrastructures enable research-practice partnership, a key facilitator to advancing both implementation practice and research (Moore & Khan, 2023; Beidas et al., 2022; Kilbourne 2020; Palinkas et al., 2016).

Existing literature suggests that implementation infrastructure is critical for high-functioning learning health systems (Grimshaw et al., 2019; Braganza et al., 2021; Kilbourne et al., 2020). Learning health systems marry innovation and discovery with implementation, and thus rely on implementation infrastructure as mechanisms to continually move evidence into practice and to scale up promising changes (Friedman, 2022). Such infrastructures include communities of practice and community of practice facilitation platforms (Brooks et al., 2024b), implementation support offices, embedded research training and mentorship programs (Grant et al., 2023; Proctor et al., 2013), and implementation laboratories (Grimshaw et al., 2019; Braganza, 2021). Implementation laboratories are one form of integrated implementation infrastructure cited specifically as learning health system enablers (Grimshaw et al., 2019; Braganza et al., 2021). Implementation laboratories are transdisciplinary teams of researchers with various applied research backgrounds including behavioural psychologists, economists, implementation scientists, intervention scientists, and any others relevant to a given implementation initiative. These teams are embedded into a given health system to study the effectiveness of various implementation approaches and inform implementation in practice based on their findings.

Implementation laboratories leverage collaborations between health delivery and research teams to establish comparative effectiveness trials and assess various implementation strategies. For example, the Audit and Feedback MetaLab in Canada has explored whether delivering audit and feedback reports to individuals or groups inspires the intended change in single sites and regional health systems (Ivers & Grimshaw, 2016). On a larger scale, the Quality Enhancement Research Initiative, an American implementation laboratory, has compiled 25 years of implementation insights to develop a roadmap of implementation strategies to improve health policy and population health outcomes (Kilbourne et al., 2019). This evidence is compelling for learning health system leaders; however, the implementation research and practice infrastructure literature is still nascent, leaving teams with little published guidance for developing such infrastructure locally (Bennett et al., 2020). Furthermore, like all implementation endeavours, context will dictate effective implementation infrastructure development.

## Study Design

### Study Purpose

This article presents a case study of practical experiences using social network analysis (SNA) to explore local context and inform integrated implementation infrastructure development. SNA is a research method that allows teams to study networks, with the goal of understanding the landscape, influential actors, clustering and the overall structure of the system in which implementation planning and execution takes place (Kim et al., 2015; Yousefi-Nooraie et al., 2015). Mapping relationships within these systems is useful for infrastructure development for numerous reasons. For example, SNA can help an organization understand their role and the role of other organizations within an existing system (Provan et al., 2005). SNA also illuminates influential actors within a system, as well as actors who act as boundary-spanners or knowledge brokers who can help move evidence into practice (Oliver, 2021), as is our aim in the field of implementation science. For implementation infrastructure development, SNA can understand relationships in a network with an eye toward enhancing capacity within the network for whatever skill (Provan et al., 2005), in our case, increasing the evidence created and used to increase the success of implementation in Alberta.

This initiative took place within a learning health system context. As such integrated infrastructure allowed for research-practice partnerships was a natural goal for implementation infrastructure development in Alberta. We document the challenges and opportunities we encountered during our SNA and offer insights on making the most of SNA results in developing implementation infrastructure.

### Study Design

SNA is a methodology that supports exploration of cross-sector collaborations to innovate, mobilize knowledge, and implement change at various system levels (Yousefi-Nooraie et al., 2022; Norton et al., 2017; Blanchet & James, 2012). Furthermore, SNA can uncover the influence of different actors, show the structure of the overall network, and identify connectors or bridges that link otherwise siloed network members (Blanchet & James, 2012). Teams use SNA in health settings to assess context, monitor relationship changes over time, and inform network alteration interventions that would positively impact care (Saatchi et al., 2023).

Our approach to SNA supplemented with qualitative interviews was designed to achieve three aims: (1) build an inventory of actors working in implementation research or practice; (2) learn how these actors currently collaborated; and (3) understand why collaborations looked the way they did (i.e., how the local system was enabling or hindering implementation research-practice collaborations). To achieve these aims, this study involved a survey to populate our inventory of people working in implementation research and practice and conduct the SNA. The study also included follow-up qualitative interviews with survey respondents to contextualize the SNA findings and learn about implementation research and practice infrastructure gaps. The detailed methods and results of the qualitative component of the study were published in early 2024 (Brooks et al., 2024a). In this article, we provide a detailed description of the survey and SNA methods and results, with enough information from the qualitative component to describe the added value of the qualitative interviews.

### Context

This article describes how a recently established intermediary, the Alberta Strategy for Patient-Oriented Research SUPPORT Unit (AbSPORU), employed SNA to explore both implementation research and implementation practice infrastructure needs at a provincial level. Intermediaries play important roles by providing implementation support and capacity-building services (Proctor et al., 2019; Metz et al., 2012). The province of Alberta has a learning health system that interfaces with numerous intermediaries, academics, health system-based researchers, and health organizations involved in implementation research and practice. Many of these organizations are interested in using implementation research to improve health outcomes, but expertise is fragmented (Brooks et al., 2024b; Flynn et al., 2021). To align and leverage expertise from across the province, AbSPORU hosted two cross-sectoral events (October 2018 and April 2019). These events garnered significant interest in building integrated infrastructure to support implementation practice and research in Alberta. In this instance, our health system partners called for increased use of implementation research to strengthen Alberta’s implementation, scale, spread and sustainment efforts. To begin the building process, event participants emphasized the importance of foundational research about our context and local needs, to ensure that the infrastructure could be designed to best capitalize on assets and address gaps. AbSPORU was well positioned to lead this work as its mandate is to strengthen evidence uptake in whatever ways made sense for local health organizations (Brooks et al., 2024b; Flynn et al., 2021).

### Survey Development and Distribution

We developed an online survey, using Qualtrics survey platform (Qualtrics, 2021), to identify organizations, offices, and individuals that (1) facilitated implementation planning and evaluation (i.e., implementation support practitioners) and/or (2) conducted implementation research (i.e., implementation researchers) in Alberta. We asked people to tell us about their workplace but to respond based on the nature of their implementation work. Respondents provided collaboration data as individuals (e.g., academic researchers, consultants), as teams (e.g., an implementation support office housed within a larger organization), or their whole organization (e.g., organizations primarily mandated to lead implementation of change). Many of the organizations in our networks were very large institutions, such as the province-wide health authority (100,000 + employees) and Alberta’s academic institutions. In large-scale organizations, there may be multiple implementers or implementation teams that never cross paths. Our approach let us collect organizational data while allowing participants to represent different levels of their workplaces. With this data, we could aggregate or disaggregate responses to visualize networks at different organization levels while honoring the reality of independent and team-based work structures in implementation.

SNA allows for analysis at the organization level while recognizing that relationships take place at the individual and team level (Provan et al., 2005). Furthermore, because implementation research and implementation support practice are not well defined in the literature, we allowed respondents to identify in what level of the organization they conducted their work. Some people worked as individual consultants, some worked within teams, some organizations are dedicated to implementation as a whole, etc. As such, we define actors as individuals, teams, and organizations, who collaborate for the purposes of conducting or studying implementation. Given the numerous ways people worked, we gave the respondents to opportunity to share where they worked within the organization but we conducted the analysis at the organization level. Having the data at all three levels allowed us to learn who were the most influential actors at different levels. It was important for us in our infrastructure efforts to learn about key individuals with whom we should connect, as well as which organizations were supporting the widest range of partnerships. Without collecting the data at the three levels, we would lose ability to see the key individuals and offices/teams within the broader organizations.

We operationalized the data collection by giving people the ability to report whether they worked as an individual, as a team within an organization, or as an organization. We then explored all the data at the lowest level provided by the survey respondents. We considered this a crude network that represented the complex reality of collaborations, but could not provide meaningful direction for our infrastructure-building initiative. We then aggregated the data to sum up the relationships within organizations across the province. For example, if an individual academic responded to the survey, they told us the name of their research lab, the department and faculty within which they worked, and their university. This data was entered into the crude dataset as is. This data was compiled with all other individuals into a department level analysis. Finally, all departmental level data within a faculty was compiled into the faculty level. Aggregating and disaggregating data required prior knowledge of the academic-health delivery ecosystem. If we had only collected data at the individual level, we would have maps with so many individuals we could not identify specific ties or key actors. Conversely, had we only collected data at the true organizational level, we would have learned which university in Alberta is involved in the most implementation partnerships, but we would not know which teams within the university were involved in the partnerships. In either case, the data was too nuanced or broad to provide our initiative meaningful direction. Thus, collecting the data at all levels and having knowledge of the overall system gave us a pragmatic ability to identify which level of the system was most valuable to inform our infrastructure-building decisions.

We focused on implementation researchers and implementation support practitioners (i.e., people who assist in rigorous implementation planning and evaluation [Metz et al., 2022; Moore & Khan, 2023]). Implementation researchers and support practitioners are likely to partner during implementation planning and evaluation (Wandersman et al., 2008). Understanding research-practice partnership dynamics was important to our partners because they saw value in developing Alberta-specific implementation research insights (Flynn et al., 2021). From a practical perspective, these two groups were also the most likely to use implementation science to inform their work. Furthermore, people at the frontlines of implementation would look to implementation support practitioners for guidance on how to implement change (Metz et al., 2022; Moore & Khan, 2023; Wandersman et al., 2008). Given our focus on implementation support practitioners as key players in implementation practice, we use the term ‘implementation support’ when discussing activities of our implementation support practitioner respondents.

We invited 145 individuals from 31 organizations in our existing contacts that we knew or suspected were eligible participants for this study. The defining feature of our exploratory SNA was that we used a mixed roster and nomination approach in our data collection. For the rostering element in the survey, we listed organizations, offices/teams, and individuals working in implementation (*n* = 133), compiled from the research team’s existing contacts. This was exploratory work which included organizations that we knew were currently active in conducting implementation support and/or implementation research. We also included organizations that we suspected were involved in these two areas of work.

### Participants

In total, 116 people accessed the survey (64.8% of all invitees). 33 respondents were ineligible and 15 provided demographic and organization information but did not indicate being involved in implementation research or practice and did not provide network data. We assumed that these 15 respondents were irrelevant to this study, given they had no network data to provide. One respondent gave two identical responses, thus we removed one. After removing the ineligible and irrelevant respondents, we had 97 potential respondents, of which 66 responded to the survey (68% of relevant invitees). The 66 respondents represented 27 organizations that conduct implementation research and/or support implementation. In the survey, respondents identified their collaborations with 54 eligible organizations involved in implementation. The respondents also indicated the nature of their work (i.e., implementation support, research, or both) and what levels of the system their implementation work took place (Table 1).

**Table 1:** Respondent characteristics

**Organization type (** ***n*** **)**		
Provincial health service delivery		2
Regional health service delivery		1
Professional organizations		3
Intermediaries		4
Community organizations		1
Research networks		3
Government		1
Primary Care		1
Embedded health services research		1
University faculties		10
**Type of implementation work (n**,**%)**		
Implementation support	6 (22.2)	
Implementation research	4 (14.8)	
Both	17 (63.0)	
**Levels of the system addressed by the respondents’ implementation work (n**,%)*		
Patient	9 (33.3)	
Practitioner	9 (33.3)	
Hospital/Unit	8 (30.0)	
Organization	8 (30.0)	
Community	9 (33.3)	
Region	9 (30.0)	
Province	7 (26.0)	

*Categories are not mutually exclusive

### Statement of University Ethics Approval

All study participants provided informed consent to be included in this research. The research design was approved by the University of Alberta Research Information Services, Research Ethics Board—Health Panel (ID: Pro00084611).

## Methods

### Survey

In the survey, we asked respondents to identify recent collaborators from the list and indicate whether they worked together for the purposes of implementation support or research. In the survey we defined implementation support as “activities involved with planning and evaluation of evidence-based healthcare to improve patient care.” Implementation research was defined in the survey as the, “study of methods to promote the integration of research findings and evidence into healthcare policy and practice.” Respondents interpreted whether they had collaborated on implementation research or support with others and indicated those ties. To be eligible to respond to the survey, respondents had to fall into one or both of these categories and be conducting their implementation research and/or support in Alberta. These eligibility criteria also acted as our network boundaries.

For the nomination element of the survey, we embedded an additional snowball sampling technique where respondents could identify additional organizations that were missing from the list (Yousefi-Nooraie et al., 2022, 2023). We invited all organizations (*n* = 11), teams (*n* = 10), and individuals (*n* = 12) identified by respondents to participate in the survey. Of these 33 potential respondents we could not find contact information for 7 potential respondents but invited the remaining 26. 19 of these 26 accessed the survey. Respondents gave additional information about the nature of their office’s implementation support or implementation research (e.g., in which sectors they worked, which levels of organizations they served). All potential respondents received three reminders to respond. The survey was open from August 2020 to January 2021.

### Social Network Analysis

We used SNA to explore the nature, distribution, and frequency of collaborations across the province’s implementation support and research communities. The responses were transformed into collaboration matrices, indicating who collaborated with whom. We removed organizations on the original list of potential collaborators from the results if (a) a representative did not respond to the survey and (b) no survey respondent indicated them as a collaborator. Respondents indicated whether they collaborated with others for implementation support and/or implementation research (organizations could fall into both). We symmetrized the data (Scott, 1991) because several actors were nominated by others but did not participate in the survey. To symmetrize the data, we replaced the missing data regarding ties (i.e., collaboration) based on the information obtained from the actors’ partners (i.e., if actor A nominated actor B, we considered there to be a collaboration between A and B regardless of whether B participated in the survey).

We developed two network maps using UCINET v6 social network analysis software (Borgatti et al., 2002) to visualize and compare the structure of implementation support and research collaborations. To populate the maps, we measured network structure, key actors, brokers, and overlap of relationships between the networks (Table 2). Specifically, we measured network density and connectedness to visualize the network structure (Luke, 2015; Carrington et al., 2005). We also calculated actor centralization and reciprocity measures to see collaboration dynamics (Yousefi-Nooraie et al., 2022). Lastly, given that organizations could fall into both implementation support and research categories, we measured the Jaccard’s coefficient, to see if and how the relationships in the two networks overlapped (Real & Vargas, 1996).

**Table 2:** Network analysis metrics

Metrics	Definitions
Density	The percentage of all possible network ties that exist (min = 0, max = 1).
Degree Centralization	Centrality is an indicator of the extent each actor collaborated with others. Centralization indicates to the inequality in the distribution of centrality in the network, ranging from 0 to 1.
Betweenness Centrality	Betweenness identifies actors that connect other actors that do not connect to each other directly. It is an indicator of bridging and brokerage.
Connectedness	The proportion of pairs of actors that could reach each other directly or indirectly (e.g., a connectedness of 0.5 means that half of the actors could be connected directly or indirectly).
Reciprocity	The percentage of all relations that are mutual (bi-directional).
Jaccard’s coefficient	The overlap between the implementation support and research networks.

### Qualitative Interviews

Once the network maps were complete, we recruited a purposive sample of the survey respondents to review the maps during qualitative interviews. We aimed to hear from people with different levels of involvement with implementation science (i.e., people who had experience working on implementation research, support, or both). We also looked for diversity in the sectors in which they worked across the research-health ecosystem (Table 3). We developed an interactive dashboard of the network maps that participants could access before and during the interviews to see where they fit in the broader networks. We also developed an interview guide based on the results of the SNA to elicit information that would contextualize the maps and help us understand the dynamics of implementation research and practice collaborations in Alberta (e.g., why organizations collaborated with some organizations but not others).

**Table 3:** Interview sample

Sectoral Focus	Number of Interviews (*n*)
Academics	6
Embedded health research teams	6
Provincial health delivery teams	3
Intermediaries	2
Regional health delivery teams	1
Government	1
Primary care delivery	1
Research networks	1
**Total interviews**	**21**

We analyzed the interviews using the Partnership Model for Research Capacity Building (Whitworth et al., 2012), which helped to identify structural and relational enablers and barriers for implementation research and practice partnerships. The full design and results of these interviews are published by Brooks et al. (2024a). We drew on the results from these interviews to inform our interpretation of the SNA results reported in the remainder of this article.

## Results

**Fig. 1:**
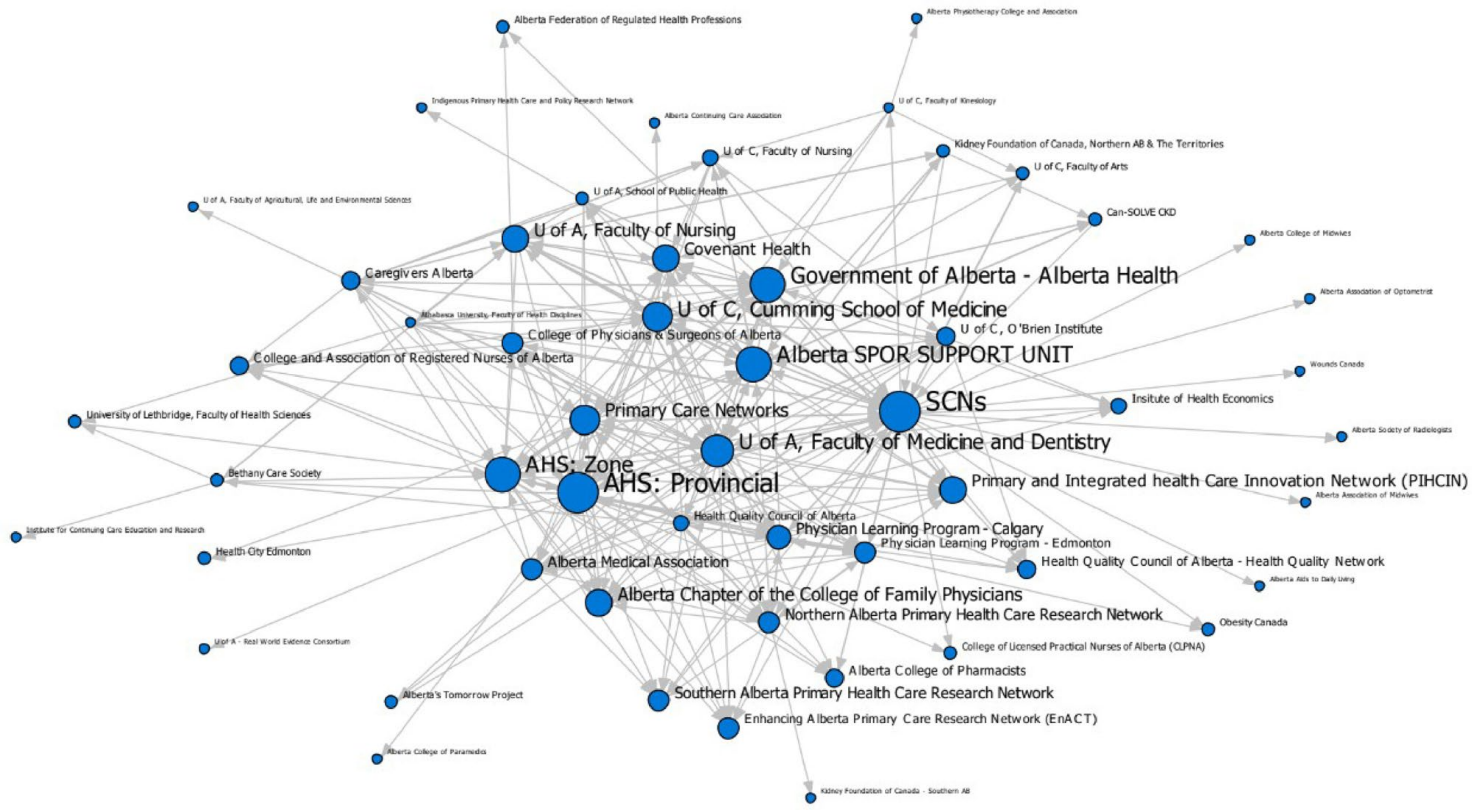
Implementation support network

**Fig. 2:**
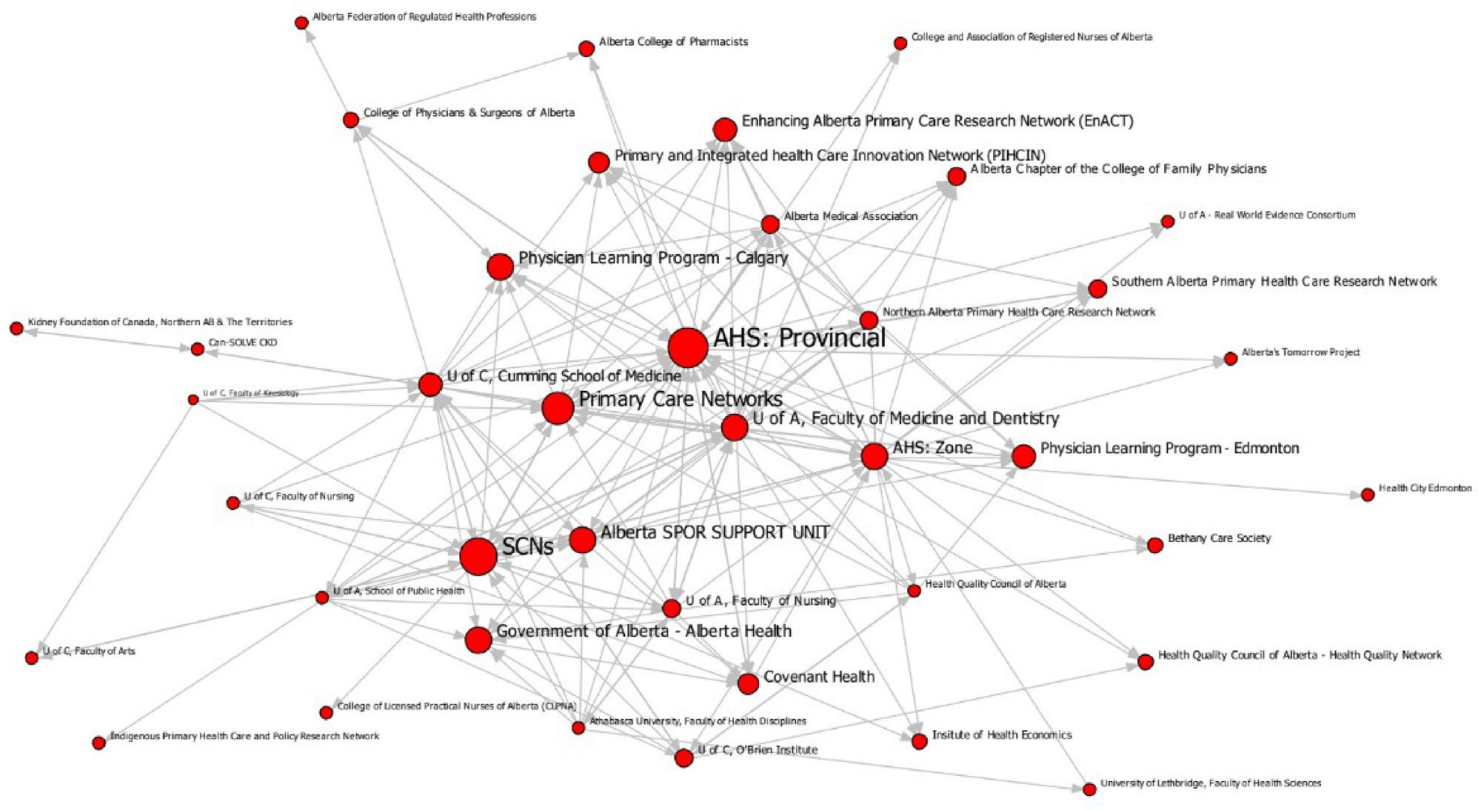
Implementation research network

The analysis generated maps pinpointing central organizational actors and insights into the collaborative dynamics of the two communities. In these maps, nodes represent organizations and the ties/arrows represent collaborations (Figs. 1 and 2). The size of the node is proportionate with the organizations’ implementation support or research collaborations (the larger the note, the more collaborations reported). A list of the organization names and abbreviations used in the maps can be found in Online Resource 1.

### Network Measurements

We measured various aspects of the two networks to see the similarities and differences between collaborations for implementation practice compared to those for implementation research (Table 4). These measurements provided insight into the network structures and collaboration characteristics of each network.

**Table 4:** Network characteristics of implementation support and research networks at the organization level

	Support Network	Research Network
Number of nodes	*n* = 54	*n =* 54
Degree Centralization	57%	42%
Density	17%	11%
Connectedness	100%	100%
Reciprocity*	27%	18%
Out degree-Centralization*	54%	34%
In degree-Centralization*	26%	24%

*Un-symmetrized network

### Network Structures

Both networks were fully connected, meaning no organizations were isolated or siloed from the rest of the network. However, this result is partially due to a methodological decision in our approach. Organizations that did not respond *and* that were not identified as collaborators were removed from the analysis as we had no data for these organizations to analyze. Nevertheless, our centrality measurements showed that two types of roles were at play to achieve this connectedness: those who were highly collaborative, and those who acted as brokers between organizations. Every organization collaborated with at least one other network member, but some organizations had far more collaborations than others. The highly collaborative organizations tended to collaborate with one another, indicating core-periphery structures in both networks. The core of the networks included prominent organizations (indicated by larger node size), which collaborated with the most partners. Meanwhile organizations with fewer collaborations made up the periphery.

### Collaboration Characteristics

The organization that had the most collaborations was Alberta Health Services, the main health service delivery organization in Alberta, which has programming both at provincial and regional levels. Alberta’s Strategic Clinical Networks (groups of embedded health research networks), AbSPORU (an intermediary), the provincial Ministry of Health, and Primary Care Networks (coordinated primary care units) were the other organizations with the most collaborations.

The implementation support network had more reciprocity (27%), where two organizations indicated collaborating with each other, than the implementation research network (18%). Furthermore, the support network was had higher density (17%) than the research network (11%). Together, these measurements indicate that the research network followed a more consultation-based approach to implementation research, where one organization requests help from others, rather than engaging in collaborative work.

**Fig. 3:**
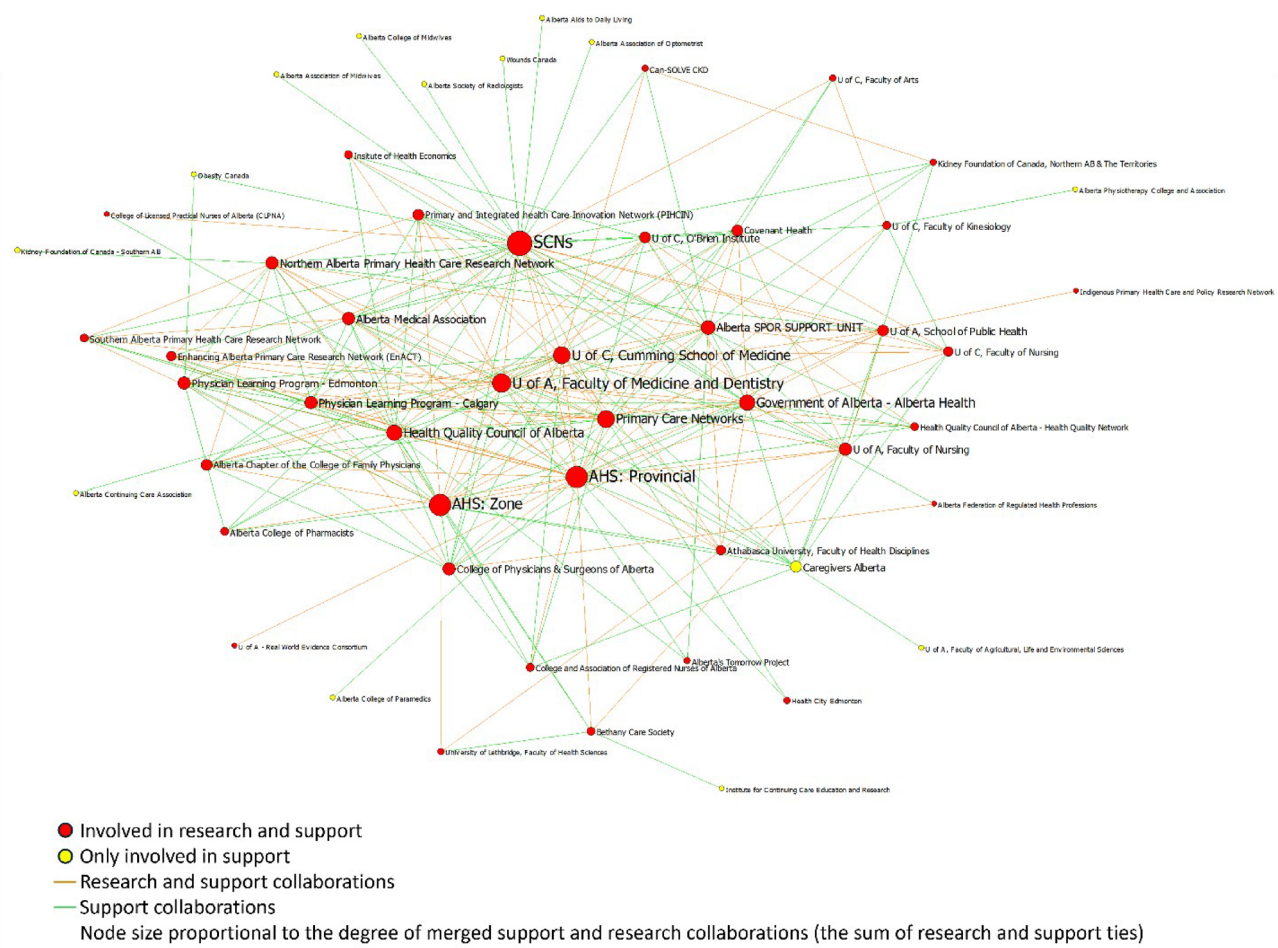
Visualization of the research-practice overlap in the support network

**Fig. 4:**
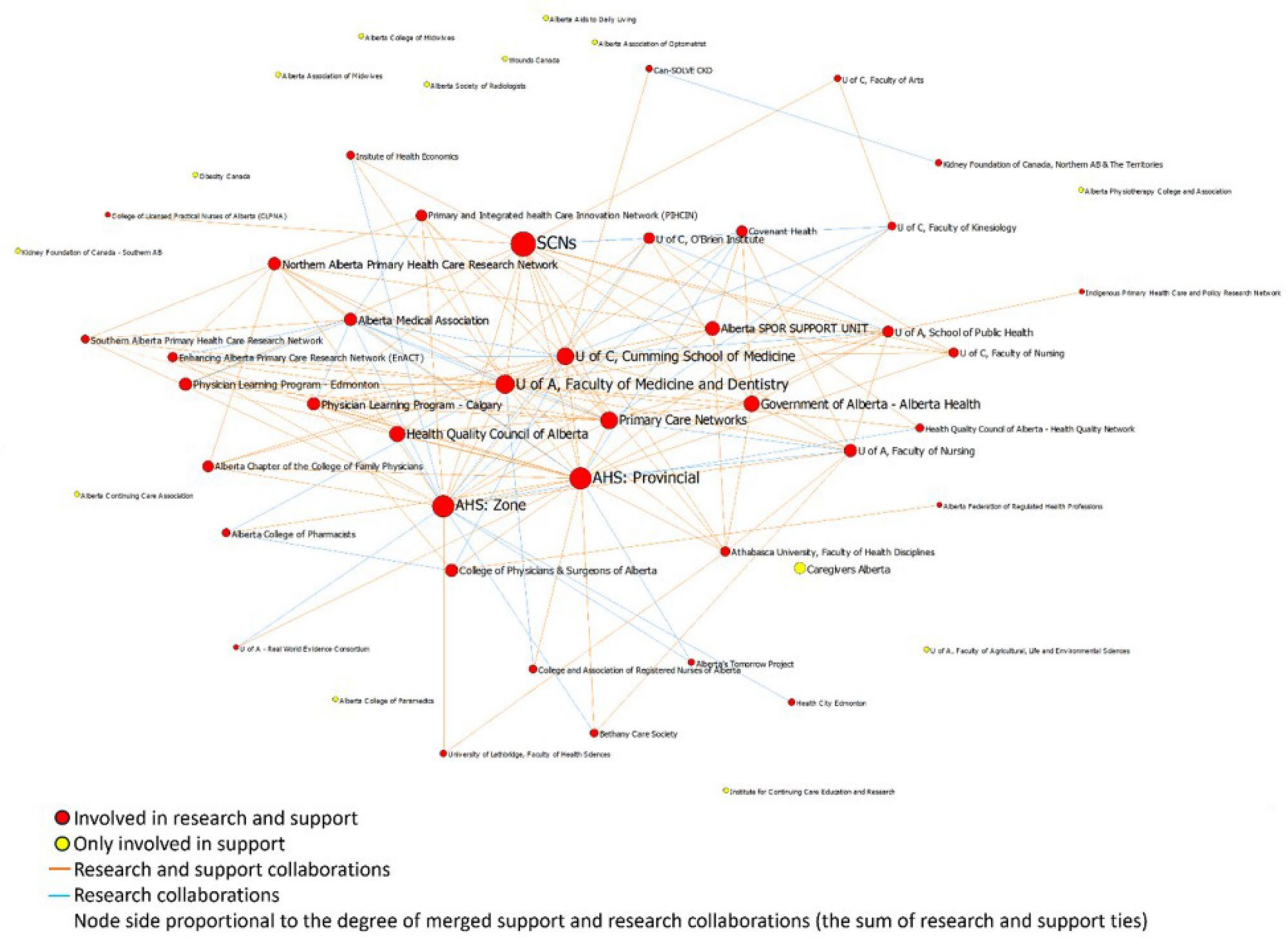
Visualization of the research-practice overlap in the research network

The Jaccard’s coefficient showed that 51% of the collaborations for implementation support and research networks overlapped, meaning that often the same organizations were involved in implementation support and research (Figs. 3 and 4). Among existing relations between organizations, 133 relationships were both support and research, 109 were only support, and 19 were only research.

A closer investigation into the characteristics of the central organizations involved in both implementation and support shows that organization type, size, and nature (i.e., academic compared to non-academic) had little bearing on an organization becoming a key playing in Alberta’s implementation research and support eco-system. However, all of the central organizations had multiple sectors of care within their mandate or led generalist care requiring referrals and collaborations with other organizations (Table 5).

**Table 5:** Characteristics of organizations central in both networks

Organizations central in both implementation research and implementation support networks				
Organization	Type	People working on implementation research and/or support (*n*)	Sector(s)	Nature of Implementation Work
Strategic Clinical Networks	Embedded health services research	50	Multiple, primarily hospital-based and specialty care	Academic
Alberta Health Services Provincial Programs	Provincial health service delivery	295	Multiple, primarily hospital-based and specialty care	Non-academic
Alberta Health Services Zones	Regional health services delivery	72	Multiple, primarily hospital-based and specialty care	Non-academic
University of Alberta Faculty of Medicine and Dentistry	University	6	Family medicine	Intermediary
University of Calgary Cumming School of Medicine	University	15	Medicine, public health	Academic
Primary Care Networks	Primary care	93	Primary Care	Non-academic
Alberta Government Ministry of Health	Government	15	Pan-spectrum	Non-academic
Health Quality Council of Alberta	Intermediary	17	Medicine/ treatment	Non-academic
AbSPORU	Intermediary	4	Multiple	Academic-non academic mixed
Alberta Medical Association	Professional organization	80	Medicine	Non-academic

### Qualitative Interviews

The full results of the qualitative interviews are reported in Brooks et al., (2024a). Using the existing Partnership Model for Research Capacity-Building (Whitworth et al., 2012), this study uncovers system-wide strengths and weaknesses that already enabled implementation research-practice partnerships. Alberta had already invested in electronic data infrastructure to support embedded health research. It also had its Strategic Clinical Networks, embedded health innovation teams that included researchers and clinician-scientists who worked together to develop, test, spread and scale healthcare improvements (Wasylak et al., 2019). However, the implementation research and support practice expertise in the system was fragmented (Brooks et al., 2024a). Additionally, there was already a culture of research-practice partnership in place but implementation-specific partnerships were far less common than partnering to develop new health interventions (Brooks et al., 2024a). This study also highlights issues that hindered implementation research-practice partnerships. Three main barriers emerged, including (1) a lack of mechanisms for implementers to connect and share implementation lessons learned, (2) inaccessible implementation research and practice training opportunities, and (3) unclear and inconsistent terminology around implementation research and practice principles (Brooks et al., 2024a).

## Discussion

### SNA as an Early Step in Exploring Implementation Infrastructure Needs

The SNA yielded important insights for AbSPORU regarding who was already involved in implementation collaborations. Our position within the system may have contributed to the richness of data input by respondents. We had a good response rate (68%), potentially indicating we were a recognized and perhaps trusted member of the broader networks we were studying. The limitations of our positionality within these networks, would emerge more through our backgrounds and biases that could have affected our qualitative analysis (Brooks et al., 2024). For this mapping study, our position as implementation scientists may have impacted our ability to capture implementation science-adjacent data (e.g., quality improvement science, change management). Nonetheless, we believe our position in the system made us a credible organization to conduct this foundational research. We were originally a knowledge translation (more commonly known as dissemination in the USA) organization in the Alberta context. We were a newcomer to the implementation science space in the province; thus we had no expectation of who the prominent actors or other network structures would be. However, the results did create surprises, which we explored through our follow-up qualitative interviews (see the next sub-section: Value-add of qualitative interviews).

From our respondents, we learned which organizations were central to the implementation support and research networks. All of these central organizations had provincial or federal funding and had mechanisms to embed either researchers or implementation support practitioners into implementation initiatives. Moreover, these players were housed in the provincial health system or in academic institutions in Alberta’s urban centers. The results also showed that the nature of implementation support in Alberta is more collaborative than implementation research, which is more consultative. Nevertheless, our results suggest that the organizations that tend to be involved in both implementation and research are those who naturally work across sectors, signaling that when trying to build initial partnerships for implementation infrastructure development, teams can consider seeking out cross-sectoral organizations first.

Growing evidence shows that the ways in which implementation support practitioners and implementation researchers work are not optimally aligned (Brooks et al., 2024a; van der Graaf et al., 2023; Stevens et al., 2020). The Jaccard’s coefficient, which measured the overlap of the networks, showed that the same individuals, teams, or organizations had capacity for both implementation support and research. This made our finding around collaborative versus consultative tendencies of the reported partnerships an unexpected nuance that deserved further investigation given the implications for partnership-building (Brooks et al. 2024a).

The SNA also highlighted who played prominent roles in the networks as highly connected or bridging organizations. This knowledge helped guide us toward actors with insights about existing implementation infrastructure and what additional infrastructure would strengthen collaborations in Alberta. Learning who was in the network peripheries was equally important to learn what, if anything, was limiting these actors’ desire or ability to collaborate for implementation. This learning was part of our foundational research prior to proto-typing implementation infrastructure for the community to consider and decide upon later. Further detail about the infrastructure co-design has been published by Brooks et al. (2024a); Flynn et al. (2021).

### The Value-Add of Qualitative Interviews

While the SNA enriched our understanding of cross-organization implementation collaborations in Alberta, the results created many questions that could not be answered through SNA measurements. For example, the 51% overlap between implementation support and research relationships was unexpected. Did this indicate that half of the time implementation research was conducted alongside the implementation of innovations? Or did respondents conflate implementation support and research when reporting on their collaborations? Why were some actors in the network so much more active in implementation support and research than others? Why did organizations collaborate with certain partners but not others? Did the respondents who did not participate in implementation research desire to incorporate implementation research into their work? If so, what was stopping them from doing so? The answers to these qualitative questions were critical for understanding what implementation research and practice infrastructures were already available and what other factors (e.g., capacity, mandates) bolstered or limited implementation collaborations. Aligning with guidance to strengthen SNA using mixed methods (Yousefi-Nooraie et al., 2018), the qualitative component of this study (Brooks et al., 2024a) increased the SNA’s value far beyond what the SNA results could generate alone. For example, we learned that Alberta has many implementation research enablers (e.g., funding streams, research teams embedded into the health system) but the province lacked a mechanism for research and practice teams to find each other, collaborate, and share lessons learned with the implementation community. We also learned about current capacity to conduct implementation research and use findings from implementation research in practice. These results informed an implementation research and practice infrastructure targeted for the needs of different implementation community members (Brooks et al., 2024a).

This SNA was the first step in several processes that underpinned the final infrastructure development. The SNA gave our team a sense of who was currently working in implementation research and support spaces. Importantly, the SNA helped us identify a sample of people working in implementation support, research or both as well as people who were well connected to the rest of the networks and those who worked in isolation. By talking with this sample in our interviews, we learned more about overarching system and what enablers and barriers impacted implementation research and support collaborations (Brooks et al., 2024a). With this information we were able to conceptualize where this infrastructure fit in the broader learning health system within the infrastructure would be located. This process is reported by Flynn et al. (2021), where they describe a two-phase process of, first, conceptualizing the infrastructure within a learning health system, then co-designing the operations of the infrastructure. Deeper description of the co-design process, and the role that intermediaries play in infrastructure facilitating co-design, is presently under review (Brooks et al., In Press in *Implementation Science Communications*).

Conducting the SNA and follow-up interviews was resource-intensive; nevertheless, completing both components helped us achieve our research aims. Moreover, presenting the SNA findings in the interviews was an excellent avenue for AbSPORU to engage implementation support practitioners and implementation researchers. The follow-up interviews with survey respondents offered an opportunity to speak with different teams about AbSPORU’s goals and services. Conducting the SNA and using the results to facilitating interviews also demonstrated AbSPORU’s commitment to strengthening implementation work in Alberta.

### Methodological Considerations

Despite the benefits of conducting the SNA and the follow-up interviews, the resources required to conduct such a study would not be readily available in many practice settings. In this sub-section we outline our resources and how these resources allowed us to conduct an SNA in Alberta, Canada. As discussed in the methods, the geography of Alberta acted as a key boundary for this SNA. However, implementation systems can occupy smaller areas (e.g., regional health systems or single site hospitals). Conversely, implementation systems can cross regional, provincial, state, and national territory lines. Our SNA focused on the organization-level for our analysis. In other contexts, collaborators might be primarily individuals, teams, larger organizations than those in our system. Exploratory SNA can support learning about implementation systems of any scale, however it is critical for research teams to have prior knowledge of the system to know what levels of analysis will be most informative. Collecting data at individual, team/office, and organization levels can help teams run various analyses and learn what type of implementation work is being conducted at different levels of the system. The first step of any SNA is to consider and set the boundaries of the networks that teams wish to explore (Blanchet & James, 2012). Teams must also match the resources available with their goals to decide what level of formality is required in their context. By formality, we mean to what degree of precision is required. For example, an SNA to inform high-risk decisions (e.g., how to break up infectious disease transmission networks) require strong and precise measures that we feel confident about. Conversely, lower risk decisions (e.g. who should we be engaging in our infrastructure building work?) can be informed with lower-resource, less precise approaches.

We conducted this study as a formal SNA (i.e., a formal piece of research), requiring resources, expertise, and university ethics board approval. To conduct our study, we hired highly qualified researchers trained in SNA and qualitative methodologies. For research software, we used Qualtrics to distribute our survey, UCINET to create our maps, and NVivo to manage and analyze our qualitative data. All these software options were subsidized by the researchers’ affiliated universities and/or purchased through grant funding. We had resources to conduct a formal SNA; however, organizations without comparable resources can employ an informal SNA approach, which uses software and methods that may not meet the standards of an academic study but still allow the team to map relationships across different organizations. With the growing interest in using SNA in community settings, other free or affordable options for SNA software (e.g., SocNetV) and web-based platforms (e.g., Kumu) have emerged. A range of qualitative research software products are also available to meet various research needs (Silver & Lewins, 2014). Rapid qualitative analysis methods (Vindrola-Padros & Johnson, 2020) also give organizations with short deadlines options to add value to their network maps. These adaptations to our approach are available for teams in resource-limited practice settings to do more informal, but equally informative, SNAs.

In addition to financial costs, the SNA and qualitative interviews required a heavy time commitment from research staff. For many on our research team, this was our first time conducting a SNA, requiring training time to understand SNA approaches. The most time-consuming task for our team was developing a survey that (1) used language about implementation research and implementation support that could be understood across sectors, (2) included a long list of potential collaborators, and (3) did not overwhelm respondents. Consulting with different implementation research and support community members helped us achieve these aims. People who helped to pilot the survey made clear that the simplicity of the survey was the most important factor. Any more than two questions, besides demographic questions, muddied the purpose of the survey and required numerous explanations of concepts. Originally, we tried to develop the survey to capture partnership nuances (e.g., Were partnerships formal or informal? Which potential collaborators did the respondents want to work with in addition to their existing collaborations?). Through our pilot, we learned that the survey should remain focused - in our case, asking about existing implementation collaborators sufficed. Further nuances can be explored in follow-up interviews. The input we received while piloting the survey was critical for our success; as such, we recommend piloting surveys with potential respondents regardless of whether you are taking a formal or informal approach to SNA.

The intention to publish the results of exploratory SNA should also be considered when deciding whether to follow a formal or informal approach. In our context, we were looking to learn who was part of the implementation support and research networks in Alberta. We invited everyone we thought might belong to the networks, which likely affected our response rate. Some potential participants may have recognized their ineligibility from the invitation, causing them not to open and respond to the online survey. We also chose to remove potential actors from our analysis if we had no data for them to analyze, which increased the appearance of connectivity and may not have shown the whole reality of the networks. We did, however, assume a collaboration between two organizations if one indicated a relationship, rather than excluding ties if both sides did not identify one another.

Given that reliability in SNA depends on adequate response rates (typically 75% or higher) (Borgatti et al., 2006), exploratory SNA creates methodological challenges for teams looking to publish their work as formal, generalizable academic studies. Purist snowball approaches, where survey respondents indicate partners rather than choosing from a list, are appropriate for exploratory SNA (Yousefi-Nooraie et al., 2022). However, this approach risks missing groups or individuals who are work disconnected from the larger network. Our team was more concerned about recall bias than response rates. To overcome recall bias, we chose to include a list of potential collaborators. We also chose to symmetrize the connectivity data, trusting that there was a partnership even if only one respondent indicated a relationship. In this study, there was no benefit for respondents to indicate untrue collaborations. It was more likely that survey respondents would forget partnerships than indicate untrue partnerships. Thus, we deliberately chose to trust there was relationship to overcome recall bias. Symmetrizing the connectivity allowed us to include relationships when indicated by just one side, instead of excluding a relationship that was not confirmed by both sides. Our wide-reaching recruitment approach and our decision to symmetrize the data may have negatively affected the reliability of this study from an academic perspective. These methodological challenges can be addressed in different ways. Using a formal versus informal SNA lens to think through the study design can help teams assess which methodological challenges they are willing to face.

### Scope Creep, for Better or Worse

We learned that studying the implementation community in an exploratory way can easily balloon in scope. We first intended simply to develop a list of implementation support practitioners and implementation researchers in Alberta. We added the SNA component to learn if and how the people on the list collaborate. The network maps illuminated the structure of implementation collaborations but could not tell us why the collaborations looked the way they did, necessitating the qualitative interviews. The data collected within the surveys was minimal. We missed opportunities to embed questions about what exactly was being transferred in implementation collaborations. We assumed that implementation research and practice knowledge was being transferred between collaborators, but we failed to ask explicitly about the contributions of each partner. Future users of SNA could consider asking about different forms of human, financial, knowledge-based or other capital that they contribute in their implementation research-practice collaborations.

Additionally, while conducting the interviews, many asked us if we would run the SNA periodically to develop a longitudinal understanding of how implementation collaborations in Alberta change over time. The longitudinal potential of SNA is one of its key strengths (Glegg et al., 2019), and would be valuable to see if and how different groups interact with new implementation infrastructure. However longitudinal studies require ongoing support. Each component of the increasing scope brings its own value but requires more resources to conduct. Given that each context will require different types of infrastructure, we recommend taking a stepwise approach to this type of foundational research, assessing if increasing the scope will add sufficient value for the resources required.

## Conclusion

This paper provides practical insights on how to use exploratory SNA to explore the state of implementation research-practice partnerships in a particular context. The results will differ based on the context in which this method is applies, but our reflections will help people with different resources to explore context either through formal or informal SNA. Specifically, our reflections have key implications for teams considering SNA as they develop implementation infrastructure. As recommended above, qualitative interviews should accompany SNA to contextualize the SNA maps. Interviews with actors in the network cores and peripheries both have valuable insights into local infrastructure needs. The survey underpinning SNA should be focused (containing one to two key questions) to maintain clarity for the respondents. Each component of our approach to this formative research requires resources and expertise. To optimize resources, teams should consider whether formal or informal SNA will help them achieve the goals of the study. In either case, teams can consider conducting SNA to inform their infrastructure initiatives as the resulting maps can be vital tools to engage people to share infrastructure needs and insights.

## Supplementary Information

Below is the link to the electronic supplementary material.

Supplementary Material 1

## Data Availability

To protect the privacy and confidentiality of the research participants, the data and materials from this study are not available.
