# Supplementary material for: Using Social Network Analysis to Inform Implementation Science Infrastructure Development
Source: Glob Implement Res Appl. 2025 Jul 31;5(4):489–504. doi: 10.1007/s43477-025-00180-8 (PMC12638342; doi:10.1007/s43477-025-00180-8)
Supplement: Supplementary file 1 — Supplementary Material 1 [file 43477_2025_180_MOESM1_ESM.docx]

**Supplement 1. Organization Names and Acronyms Used in Figures**

AHS: Provincial – Alberta Health Services, Provincial Initiatives

AHS: Zone – Alberta Health Services, Zone Initiatives

Alberta Medical Association

Alberta SPOR SUPPORT UNIT – Alberta Strategy for Patient Oriented Research SUPPORT Unit

Athabasca University, Faculty of Health Disciplines

Bethany Care Society

Can-SOLVE CKD - Canadians Seeking Solutions and Innovations to Overcome Chronic Kidney Disease Network.

Caregivers Alberta

College of Physicians & Surgeons of Alberta

Covenant Health

Government of Alberta - Alberta Health

Health Quality Council of Alberta

Kidney Foundation of Canada, Northern AB (Alberta) & The Territories

Northern Alberta Primary Health Care Research Network

Physician Learning Program - Calgary

Physician Learning Program - Edmonton

Primary Care Networks

SCNs – Strategic Clinical Networks

U of A (University of Alberta), Faculty of Medicine and Dentistry

U of A (University of Alberta), Faculty of Nursing

U of A (University of Alberta), School of Public Health

U of C (University of Calgary), Cumming School of Medicine

U of C (University of Calgary), Faculty of Arts

U of C (University of Calgary), Faculty of Kinesiology

U of C (University of Calgary), Faculty of Nursing

U of C (University of Calgary), O'Brien Institute

University of Lethbridge, Faculty of Health Sciences
